# The Clinical, Symptom, and Quality-of-Life Characterization of a Well-Defined Group of Patients with Posttreatment Lyme Disease Syndrome

**DOI:** 10.3389/fmed.2017.00224

**Published:** 2017-12-14

**Authors:** Alison W. Rebman, Kathleen T. Bechtold, Ting Yang, Erica A. Mihm, Mark J. Soloski, Cheryl B. Novak, John N. Aucott

**Affiliations:** ^1^Department of Medicine, Johns Hopkins University School of Medicine, Baltimore, MD, United States; ^2^Department of Physical Medicine and Rehabilitation, Johns Hopkins University School of Medicine, Baltimore, MD, United States

**Keywords:** Lyme disease, signs and symptoms, quality of life, case series, posttreatment Lyme disease syndrome

## Abstract

**Background:**

The increased incidence and geographic expansion of Lyme disease has made it the most common vector-borne infection in North America. Posttreatment Lyme disease syndrome (PTLDS) represents a subset of patients who remain ill following standard antibiotic therapy for Lyme disease. The spectrum of symptoms and their impact on quality of life remain largely unexplored among patients with well-documented PTLDS.

**Objective:**

To characterize a case series of patients with well-documented PTLDS compared to a sample of healthy controls.

**Methods:**

Sixty-one participants met the proposed case definition for PTLDS. Twenty-six healthy controls had neither a clinical history of Lyme disease nor current antibodies to *Borrelia burgdorferi*. Participants with PTLDS and controls were evaluated by physical exam, clinical laboratory testing, standardized questionnaires, and a 36-item current symptom list.

**Results:**

Compared to controls, participants with PTLDS reported significantly greater fatigue, pain, sleep disturbance, and depression (Fatigue Severity Scale: 50.0 ± 10.6 vs. 19.8 ± 8.6; Short-Form McGill Pain Questionnaire: 13.7 ± 8.3 vs. 0.8 ± 1.9; Pittsburgh Sleep Quality Index: 10.1 ± 4.7 vs. 4.1 ± 2.1; Beck Depression Inventory-II: 15.1 ± 7.7 vs. 2.2 ± 3.2; *p* < 0.001 for each), and significantly lower quality of life (SF-36 Physical Component Score: 33.9 ± 9.7 vs. 55.1 ± 6.2; Mental Component Score: 42.9 ± 10.1 vs. 54.2 ± 5.4; *p* < 0.001 for each). Nineteen non-PTLDS-defining symptoms were found to be significantly more severe among participants with PTLDS than controls, including sleep difficultly and visual complaints. Initial delayed or misdiagnosis was characterized in 59.0% of participants with PTLDS, and 32.2% had abnormal vibratory sense.

**Conclusion:**

Although physical exam and clinical laboratory tests showed few objective abnormalities, standardized symptom questionnaires revealed that patients with PTLDS are highly and clinically significantly symptomatic, with poor health-related quality of life. PTLDS patients exhibited levels of fatigue, musculoskeletal pain, sleep disturbance, and depression which were both clinically relevant and statistically significantly higher than controls. Our study shows that PTLDS can be successfully identified using a systematic approach to diagnosis and symptom measurement. As the prevalence of PTLDS continues to rise, there will be an increased need for physician education to more effectively identify and manage PTLDS as part of integrated patient care.

## Introduction

Lyme disease was first recognized in the United States in the mid-1970s and the tick-borne bacterial pathogen, *Borrelia burgdorferi*, was first identified in 1982 (1). Since then, the infected tick vectors and the disease have spread geographically in North America (2, 3). Incidence rates have continued to rise in recent years, and the CDC currently estimates approximately 300,000 new cases annually in the United States (4).

The distinctive feature of early Lyme disease is the erythema migrans rash, which is recognized in many, but not all patients an average 7–10 days after the bite of an infected tick (5, 6). The rash may be accompanied by viral-like symptoms such as fever, myalgia, arthralgia, or fatigue. Treatment of early Lyme disease is generally effective in resolving objective manifestations of the disease and preventing development of later manifestations of untreated infection (7). Since the rash is not always present or recognized in early Lyme disease and the viral-like symptoms can mimic other illnesses, delayed diagnosis and treatment remain a significant problem (8). In untreated Lyme disease, the bacteria may disseminate through the blood stream to other areas of the skin, nervous system, or heart (5). Late, untreated disease can present weeks to months after the initial infection, and primarily involves the joints and the nervous system (5, 9), and can be more difficult to treat.

In contrast to untreated stages of infection, it has been noted that approximately 10–20% of patients experience a constellation of prolonged, primarily patient-reported symptoms following standard antibiotic treatment for early or late Lyme disease (10). This chronic illness, most recently called posttreatment Lyme disease syndrome (PTLDS), is distinct from the slowly resolving, self-limited symptoms sometimes seen after completion of antibiotic therapy (11). It is also a defined subset of patients distinct from those with non-specific chronic Lyme disease, which has become a focal point of ongoing controversy and debate (11). The pathophysiology of PTLDS is currently unknown, with additional research needed to delineate potential roles of infection-induced immune dysfunction (12, 13), inflammation due to persistent bacteria (14) or bacterial debris (15), or other mechanisms. Risk factors for PTLDS have been identified which include delay in diagnosis (16, 17) and increased severity of initial illness, including the presence of neurologic symptoms (7, 18). Moreover, initial exposure to non-recommended antibiotics (19) or steroids (20) has also been associated with worse clinical outcomes.

In 2006, the Infectious Diseases Society of America (IDSA) published a proposed case definition for PTLDS (21). Briefly, this definition relies on prior physician-documented Lyme disease, treatment with standard of care antibiotics, and the development of significant fatigue, widespread musculoskeletal pain, and/or cognitive difficulties that last for a period of at least 6 months, and began within 6 months of a Lyme diagnosis and recommended treatment (21). Patients can be excluded for a range of symptoms, conditions, or laboratory abnormalities that could be associated with or explain the symptomatology of PTLDS (21). As antibodies to *B. burgdorferi* are known to fall over time in treated patients regardless of clinical outcome (7, 22–24), and convalescent antibodies after treatment of early Lyme disease are positive in 65% of patients (25), presence of reactive antibodies is not part of this definition. In the clinical setting, PTLDS is largely an historical diagnosis that hinges on the ability to document symptom onset after an episode of physician-documented Lyme disease that was adequately treated with standard of care antibiotics.

Patients with PTLDS represent a defined subset of a heterogeneous group of patients evaluated for unexplained fatigue, pain, and neurocognitive symptoms by primary care and sub-specialty physicians. However, the clinical, laboratory, and symptom characteristics of PTLDS remain largely unexamined and unreported in the literature, particularly among patients whose initial Lyme disease diagnosis and treatment reflects the community practice setting. To our knowledge, no studies have described patients with rigorously defined PTLDS drawn from a community population. The goal of the current study is to delineate PTLDS-specific patterns in physical exam findings, clinical laboratory results, symptom reporting, and quality of life by characterizing the first 61 participants enrolled in an ongoing case series of well-documented PTLDS, and to compare this group to a sample of control participants screened for a history of prior Lyme disease.

## Materials and Methods

### Study Participants

Participants with PTLDS were physician or self-referred to the Johns Hopkins Lyme Disease Research Center and were subsequently invited to participate in this study, or self-referred to the Center specifically for research participation. The screening process and eligibility criteria are detailed in Table 1. We estimate that approximately 16% of patients referred to the center were eligible for and chose to enroll in this study.

**Table 1:** Eligibility criteria for study enrollment.

Medical record-confirmed inclusion criteria	
Confirmed or probable history of physician-documented Lyme disease (26)	(a) Evidence of physician-documented erythema migrans rash OR (b) Evidence of new-onset objective finding (oligoarthritis with joint swelling, facial palsy, neuropathy, meningitis, encephalitis, or carditis), and concurrent laboratory evidence of infection performed by a laboratory following CDC recommendations for test interpretation (27) OR (c) Evidence of new-onset symptoms not attributable to other cause with concurrent laboratory evidence of infection performed by a laboratory following CDC recommendations for test interpretation (27)
Appropriate antibiotic treatment	Evidence of recommended antibiotic dose and duration for treatment of early or late Lyme disease^a^
Posttreatment symptoms associated with Lyme disease exposure	Evidence of at least one of the following; fatigue, musculoskeletal pain, or neurocognitive complaints, that began within 2 years of Lyme disease diagnosis^b^

**Self-report inclusion criteria**	
Severity of posttreatment symptoms	At least one symptom experienced in the past 2 weeks that limits daily functioning at least half of the time when present

**Self-report exclusion criteria**	
Physician diagnosis ever	Lyme vaccine, sleep apnea or narcolepsy, any autoimmune disorder, cirrhosis or hepatitis B/C, HIV, dementia, schizophrenia, bipolar illness, delusional disorder, major depression
Physician diagnosis prior to onset of Lyme disease	Fibromyalgia, chronic fatigue syndrome, unspecified chronic neurologic disease, unexplained chronic pain
Physician diagnosis in past two years	Cancer or malignancy
In past year	Illicit substance use, use of marijuana ≥3 times a week, use of prescription drugs not as prescribed
Current	CAGE alcoholism screen (28), pregnancy

*^a^Considered any of the following: Doxycycline 100 mg BID for ≥10 days, Amoxicillin 500 mg TID ≥ 14 days, Ceftin 500 mg BID ≥ 14 days, Ceftriaxone 2 g Q24 ≥ 14 days. In addition, Tetracycline 500 mg TID for ≥14 days and Augmentin 875 mg BID ≥ 14 days were also permitted as recommended dose and durations; however, no participants were enrolled with these regimens*.

*^b^In order to increase specificity and conform to the Infectious Diseases Society of America definition, the current analysis was limited to (a) participants whose posttreatment Lyme disease syndrome (PTLDS) symptoms began within 6 months of diagnosis and (b) participants whose PTLDS symptom had lasted for 6 months or longer at time of study enrollment (21)*.

Interested patients were provided study information and screened for self-reported exclusionary medical conditions diagnosed within the time frames indicated. If eligible, participants gave approval for medical record review of prior Lyme disease diagnoses, signs, symptoms, two-tier serologic test results, and antibiotic treatment. Patients were considered eligible if the medical record documented prior “confirmed” (LD-confirmed) or “probable” (LD-probable) early or late disease, following the CDC’s 2011 case classification (26). All serologic tests for antibodies to *B. burgdorferi* were considered confirmatory in the context of illness duration, and if conducted at a laboratory following CDC recommendations for test interpretation (26, 27).

Healthy controls were originally recruited from an internal medicine practice in the same geographic location as part of a separate longitudinal cohort study. They did not have a clinical history suspicious for diagnosed or undiagnosed Lyme disease, and were CDC-negative on two-tier testing for antibodies to *B. burgdorferi*. Controls were screened for the same self-reported exclusionary medical conditions found in Table 1, with the exception of the illicit substance and prescription drug abuse questions.

All participants with PTLDS and controls were over the age of 18 at time of study enrollment, and both groups were enrolled throughout the calendar year. This study was approved by the Institutional Review Board of the Johns Hopkins University School of Medicine and written consent was obtained from all participants. All subjects gave written informed consent in accordance with the Declaration of Helsinki. The protocol was approved by the Institutional Review Board of the Johns Hopkins University School of Medicine.

### Clinical and Laboratory Evaluation

A trained interviewer administered a series of detailed questionnaires concerning general medical, Lyme disease-specific, medication, and symptom histories. A physical exam was performed that included measurement of respiratory rate, pulse, and blood pressure with the participant in the prone position and again after 10 min of standing. The lymph nodes, thyroid, pharynx, lung, heart, liver span, and spleen were all examined. A complete joint exam was performed to assess for evidence of joint inflammation or synovitis. An extensive neurologic assessment was performed that included cranial nerve, motor, cerebellar, and sensory exams. Vibratory index was measured on the distal interphalangeal joint of the index finger and on the interphalangeal joint of the hallux using a Rydel-Seiffer 64 Hz tuning fork (29).

A complete blood count, complete metabolic count, C-reactive protein, enzyme-linked immunosorbent assay, and immunoglobulin M and immunoglobulin G Western Blot (IgM-WB/IgG-WB) testing for antibodies to *B. burgdorferi* were obtained. All Lyme serologic tests were performed through a large commercial laboratory following CDC recommendations for test interpretation (27).

### Symptoms and Quality-of-Life Evaluation

Symptoms were measured by standardized questionnaires including the Fatigue Severity Scale (FSS), the Short-Form McGill Pain Questionnaire (SF-MPQ), the Pittsburgh Sleep Quality Index (PSQI), and the Beck Depression Inventory II (BDI). These standardized questionnaires are widely used to measure fatigue, pain, sleep quality, and depression in clinical and research settings. Specifically, the FSS 9-item fatigue metric has summary scores ranging from 9 to 63 with a higher score indicating worse fatigue, and with ≥36 indicating clinically relevant levels of fatigue (30). The SF-MPQ 15-item pain metric has summary scores ranging from 0 to 45 with a higher score indicating worse pain, and with ≥4 indicating a clinically significant experience of pain (31, 32). The PSQI sleep metric has summary scores ranging from 0 to 21 with a higher score indicating worse sleep quality, and with ≥6 indicating clinically significant poor sleep quality (33). The BDI 21-item depression metric has summary scores ranging from 0 to 63 with a higher score indicating worse depression, and with ≥14 indicating mild, moderate, or severe depression (34).

Additionally, participants were asked to self-administer a 36-symptom list developed based on prior clinical and research experience among patients with PTLDS. For each of the 36 symptoms, participants indicated presence and severity (“absent,” “mild,” “moderate,” or “severe”) over the past 2 weeks.

Quality of life was measured by the Short-Form Health Survey, Version 2 (SF-36). This 36-item quality-of-life metric can be summarized into Physical and Mental Component Scores (PCS and MCS, respectively), with higher score indicating higher quality of life (35). These scores can also be compared with the US population mean (50.0 ± 10.0) (35).

### Statistical Analyses

First, we summarized all participants’ demographic characteristics. For participants with PTLDS, we then summarized their Lyme disease history. We then compared participants with PTLDS and healthy controls on their laboratory results and physical exam findings. Summary scores from standardized questionnaires measuring symptoms and quality of life were plotted by group, compared with clinically relevant cutoffs and/or the population mean, and contrasted between participants with PTLDS and controls. For the 36-symptom list, we compared participants with PTLDS and controls, and extracted the symptoms that were statistically different between the two groups. We then plotted the proportion of participants reporting “mild,” “moderate,” or “severe” on these differentiating symptoms. For each symptom, we also calculated the difference in the proportion reporting a severity of “moderate” or above between participants with PTLDS and controls. All analyses were then repeated without controls to compare LD-confirmed with LD-probable cases.

Counts and percentages were used to summarize categorical data. Means and SDs or medians and interquartile ranges (IQR) were used to summarize continuous variables, as appropriate. All comparisons were performed using *t*-tests or non-parametric Wilcoxon rank sum tests for continuous variables, and chi-square or Fisher’s exact tests for categorical variables, as appropriate. Regression analyses were conducted where additional variables were introduced as potential confounders. Specifically, we used multiple linear regression models for the original continuous outcome variables, as well as logistic regression models for outcome variables dichotomized based on normal cutoff values. The predictor of interest was group indicator (PTLDS vs. control group). Age, gender, and blood pressure medication use were adjusted for where appropriate. A *P*-value less than 0.05 was considered significant. All statistical analyses and graphs were performed using SAS (version 9.3, SAS Institute, Cary, NC, USA), R (version 3.3.1), and GraphPad Prism (version 6.05, La Jolla, CA, USA).

## Results

### Participant Characteristics

The PTLDS and control participant groups were similar by gender (PTLDS: 52.5% female vs. controls: 53.9%, *p* = 0.906), and race (PTLDS: 93.4% white, non-Hispanic vs. controls: 88.5%, *p* = 0.422). Although not statistically significant, the PTLDS group was a mean of 5.4 years younger (PTLDS: 49.3 vs. controls: 54.7, *p* = 0.157). A significantly higher proportion of controls reported graduate or professional training (PTLDS: 24.6% vs. controls: 61.5%, *p* = 0.001). Among participants with PTLDS, 3 (4.9%) were currently receiving and 7 (11.5%) had ever received disability related to Lyme disease.

### Lyme Disease History

The Lyme disease history of the PTLDS group is described in Table 2. When the first signs or symptoms of Lyme disease appeared, the majority (95.1%) were residents of states which accounted for 96% of all confirmed case reports in 2014 (3). Based on medical record review, 68.9% met the CDC surveillance criteria for LD-confirmed at the time of their initial presentation prior to treatment (26). The remaining 31.1% met criteria for LD-probable. Our cohort was a median of 3.6 years from onset of PTLDS symptoms to study enrollment, with a range of 8.3 months to 27.7 years. Time from illness onset to first recommended course of antibiotic treatment was a median of 30 days. Based on self-report, the median total length of antibiotic exposure at time of enrollment was approximately 3 months. As defined in Table 2, delayed or initial misdiagnosis could be characterized in 59% of the sample.

**Table 2:** Lyme disease history of 61 participants with posttreatment Lyme disease syndrome (PTLDS).^a^

	PTLDS (*n* = 61)
State of residence^b^ Maryland Pennsylvania Delaware New Jersey One participant enrolled from each of the following: Virginia, W. Virginia, Washington DC, Connecticut, Vermont, Kentucky	40 (65.6%) 10 (16.4%) 3 (4.9%) 2 (3.3%) 6 (9.8%)

Medical record-confirmed Lyme disease presentation CDC confirmed (26) Physician-documented erythema migrans rash^c^ Early objective finding/(+) enzyme-linked immunosorbent assay (ELISA)/WB^d^ Late objective finding/(+) IgG-WB^e^ CDC probable (26) Viral-like illness/(+) ELISA/WB Non-acute patient reported symptoms/(+) IgG-WB^f^	34 (55.7%) 2 (3.3%) 6 (9.8%) 5 (8.2%) 14 (23.0%)

Observed/removed tick in month prior to onset	10 (16.4%)

Duration of illness from onset of PTLDS symptoms to enrollment	3.6 years [1.9–9.9 years] (8.3 months–27.7 years)

Duration of illness from onset of first sign/symptom to start of first recommended antibiotic treatment course^g^	30 days [3.0 days, 7.0 months] (0 days–3.4 years)

Non-recommended antibiotics prior to recommended antibiotics^g^	7 (11.5%)

Steroids prior to recommended antibiotics^g^	6 (9.8%)

Total antibiotic exposure from symptom onset	2.9 months [1.6, 6.6 months] (14 days, 3.2 years)

Delayed or misdiagnosis^h^	36 (59.0%)

*^a^Presented as *n* (%) for categorical variables or median [interquartile range] (minimum − maximum) for continuous variables*.

*^b^Defined as state of residence when first evidence of Lyme disease appeared, unless self-reported clear exposure elsewhere*.

*^c^Of these, 18 (52.9%) also had a positive two-tier serology, 1 (2.9%) also had Bell’s Palsy, 1 (2.9%) also had neuropathy, and 2 (5.9%) also had carditis*.

*^d^One patient with carditis and positive ELISA/IgM-WB, and one patient with Bell’s Palsy and positive ELISA/IgM-WB*.

*^e^Five patients with late Lyme arthritis and positive IgG-WB, and one patient with neuropathy and positive IgG-WB*.

*^f^Of these, 12 with positive IgG-WB, 1 with positive C6 antibody, and 1 with both positive IgG-WB and C6 antibody*.

*^g^Recommended antibiotic regimens were considered any of the following: Doxycycline 100 mg BID for ≥10 days, Tetracycline 500 mg TID for ≥14 days, Amoxicillin 500 mg TID ≥ 14 days, Augmentin 875 mg BID ≥ 14 days, Ceftin 500 mg BID ≥ 14 days, Ceftriaxone 2 g Q24 ≥ 14 days. Other drug, or lower dose or durations were considered non-recommended antibiotic regimes*.

*^h^Defined as either (a) duration ≥30 from first sign/symptom to first recommended antibiotic treatment, (b) exposure to non-recommended antibiotics prior to recommended antibiotics, or (c) exposure to steroids prior to recommended antibiotics*.

### Clinical and Laboratory Evaluation

Clinical laboratory and physical exam findings are shown in Table 3. At the time of the study visit, 43.3% of participants with PTLDS had a positive IgG-WB. We also examined participants’ lymphocyte count and liver function tests. Although the proportion of participants with PTLDS with lymphocyte and liver function abnormalities was higher than controls for each (lymphocyte count: 3.4 [2/59] vs. 0.0%, AST: 8.3 [5/60] vs. 7.7%, ALT: 20.0 [12/60] vs. 7.7%), no statistically significant differences were found, even after controlling for age.

**Table 3:** Laboratory markers and physical exam findings of 61 participants with posttreatment Lyme disease syndrome (PTLDS) and 26 controls.^a^

	PTLDS (*n* = 61)	Controls (*n* = 26)	*p*-Value
Two-tier serology at study visit Positive/equivocal ELISA Positive IgM-WB Positive IgG-WB	34/60 (56.7%) 7/60 (11.7%) 26/60 (43.3%)	1 (3.8%) 0 (0.0%) 0 (0.0%)	<0.001 0.096 <0.001

Other laboratory values Absolute lymphocyte count, 10^3^ µL AST, U/L ALT, U/L C-reactive protein, mg/L	1.9 [1.6–2.2] (0.7–3.1) 22 [17–28] (12–112) 23 [15–36] (5–204) 0.1 [0.1–0.3] (0–6.6)	1.8 [1.4–2.2] (1.1–2.8) 22 [19–27] (16–46) 20 [16–24] (11–78) ND	0.634 0.628 0.497 N/A

Physical exam Respiratory rate (sitting) Pulse (sitting) Systolic blood pressure (sitting) Diastolic blood pressure (sitting) Met criteria for hypertension^b^ Spleen tip present Lung auscultation abnormal Thyroid abnormal Pulse change (laying to 10-min standing) Met criteria for POTS^c^	16 [14–16] (12–20) 69 [65–75] (48–120) 133 [117–145] (92–179) 88 [79–93] (63–108) 32/60 (53.3%) 1 (1.6%) 1 (1.6%) 1 (1.6%) 9 [6–16] (−26 to 55) 2/59 (3.4%)	13 [12–14] (12–14) 69 [60–72] (50–84) 128 [121–140] (106–160) 78 [73–85] (61–90) 9/25 (36.0%) 0/25 (0.0%) 0/25 (0.0%) ND ND ND	<0.001 0.714 0.718 0.003 0.145 1.000 1.000 N/A N/A N/A

Neurologic exam Cranial nerve 2–12 abnormal Motor exam abnormal Vibration sense abnormal^d^ Sensory (monofilament) abnormal Pin abnormal Proprioception abnormal Right and/or left foot stand abnormal Finger tap abnormal Rhomberg abnormal	6 (9.8%) 1/59 (1.7%) 19/59 (32.2%) 6 (9.8%) 3 (4.9%) 3 (4.9%) 7/59 (11.9%) 1/60 (1.7%) 1/59 (1.7%)	0/25 (0.0%) 0/25 (0.0%) ND ND ND ND ND ND ND	0.175 1.000 N/A N/A N/A N/A N/A N/A N/A

Cardiac exam abnormal Joint exam abnormal	2 (3.3%) 10/60 (16.7%)	0/25 (0.0%) 1/25 (4.0%)	1.000 0.163

*^a^Presented as *n* (%) for categorical variables and median [inter-quartile range] (minimum − maximum) for continuous variables. ND = data not available for control sample*.

*^b^Systolic blood pressure ≥140 mmHg and/or diastolic blood pressure ≥90 mmHg*.

*^c^Postural orthostatic tachycardia syndrome, heart rate >120 bpm standing, or increase in pulse ≥30 bpm from laying to 10-min standing (36)*.

*^d^Below age-adjusted normal vibration threshold values (29) in either upper (distal interphalangeal joint of the index finger) or lower (interphalangeal joint of the hallux) extremities on either right or left side using a Rydel-Seiffer 64 Hz tuning fork*.

None of the participants with PTLDS were found to have lymphadenopathy, hepatomegaly, or pharyngeal abnormalities on examination. Participants with PTLDS had a median respiratory rate three breaths per minute higher (*p* < 0.001), and their median diastolic blood pressure was 10 mmHg higher than controls (*p* = 0.003). These results remained similar after controlling for age, gender and current blood pressure-lowering medication use. On the neurologic exam, we found a minority of participants with cranial nerve, motor, or cerebellar abnormalities, as well as 32.2% with scores below age-adjusted cutoffs for vibratory sense in upper or lower extremities (29). Although the proportion with cardiac or joint abnormalities was higher among participants with PTLDS compared to controls, these differences were not statistically significant.

### Symptoms and Quality-of-Life Evaluation

**Figure 1:**
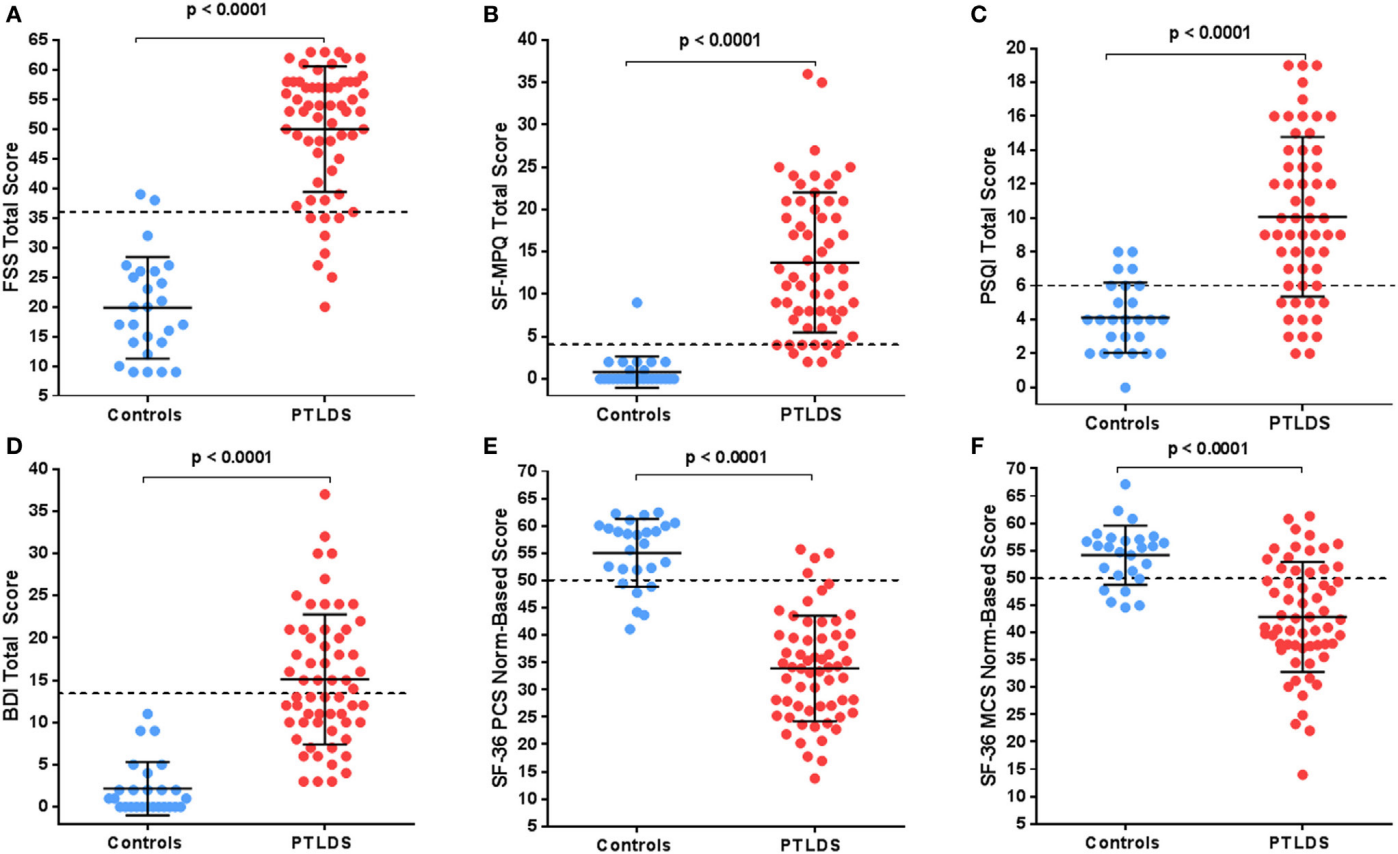
Participants with posttreatment Lyme disease syndrome (PTLDS) were compared to controls on the following: **(A)** Fatigue Severity Scale, **(B)** the Short-Form McGill Pain Index, **(C)** Pittsburgh Sleep Quality Index, **(D)** the Beck Depression Inventory-II, and the SF-36 **(E)** Physical and **(F)** Mental norm-based scores. The mean and 1 SD are shown by the solid lines. Clinically relevant cutoffs for each measure are shown by the dotted line.

Figures 1A–D compare results of standardized symptom questionnaires among participants with PTLDS and controls. The PTLDS group reported significantly higher levels of fatigue, pain, poor sleep quality, and depression than controls (FSS: 50.0 ± 10.6 vs. 19.8 ± 8.6; SF-MPQ: 13.7 ± 8.3 vs. 0.8 ± 1.9; PSQI: 10.1 ± 4.7 vs. 4.1 ± 2.1; and BDI: 15.1 ± 7.7 vs. 2.2 ± 3.2; *p* < 0.001 for each). The proportion reporting symptom severity above clinically relevant cutoffs are significantly higher in the PTLDS group (FSS: 86.9 vs. 7.7%; SF-MPQ: 93.2 [55/59] vs. 3.8%; PSQI: 79.3 [46/58] vs. 26.9%; BDI: 50.0 [30/60] vs. 0.0%; *p* < 0.001 for each).

**Figure 2:**
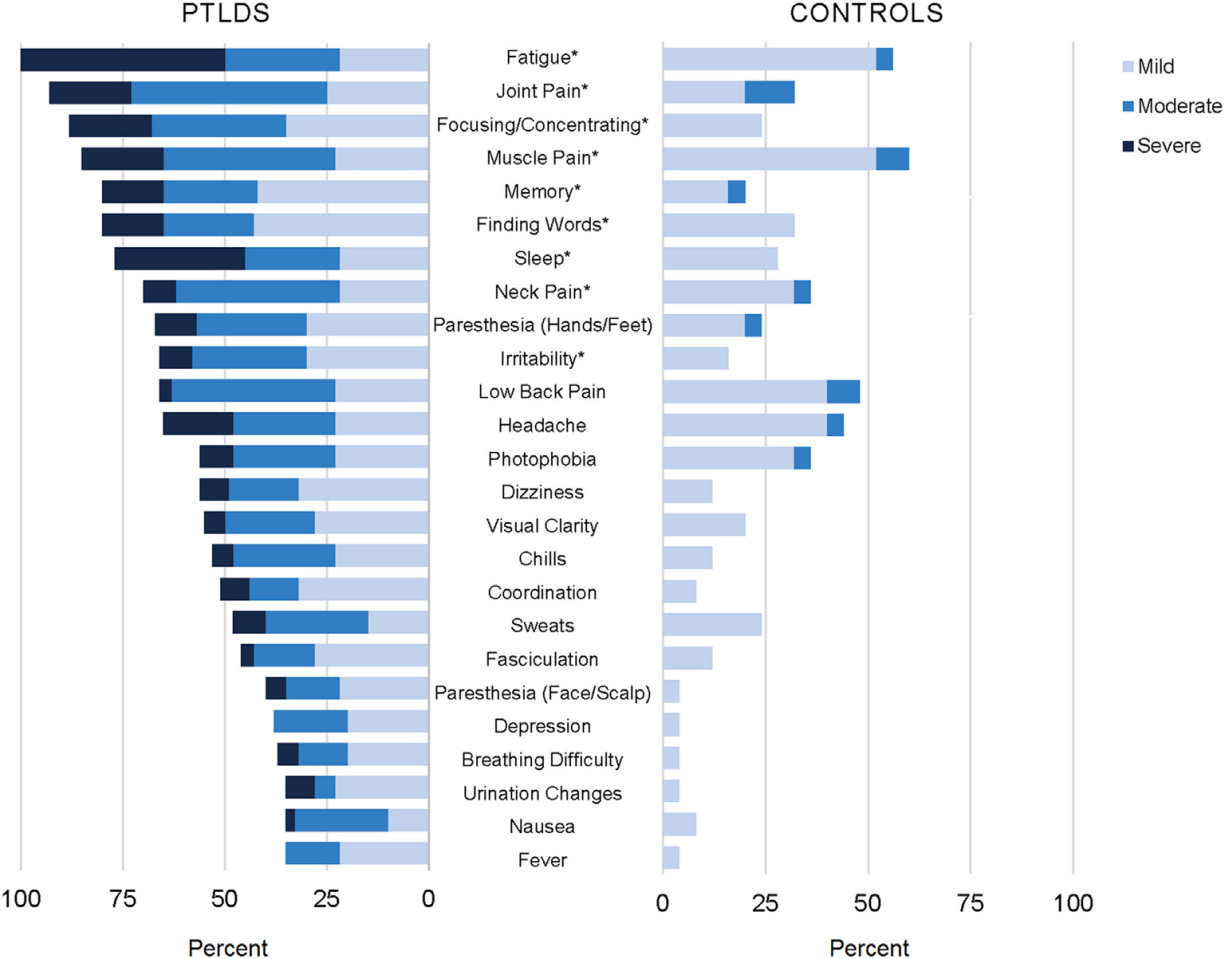
Participants with posttreatment Lyme disease syndrome (PTLDS) and controls were asked about presence and severity of 36 signs/symptoms over the past 2 weeks. Displayed are the 25 signs/symptoms with a statistically significant difference in severity by group (*p* < 0.05), ordered by frequency within the PTLDS group. The nine signs/symptoms with a statistically significant difference at the *p* < 0.001 level are indicated with an asterisk.

Twenty-five of the 36 symptoms included on our list were found to be significantly different between cases and controls (*p* < 0.05; see Figure 2). Conversely, joint swelling, double vision, drooping facial muscle, drooping eyelid, tinnitus, heart palpitations, swollen lymph nodes, vomiting, diarrhea, sore throat, and anxiety were not found to be statistically different for participants with PTLDS compared to controls. The ten symptoms with the highest difference in proportion reporting “moderate” or above by group were (in descending order); fatigue, joint pain, sleep difficulty, muscle pain, focusing/concentrating, neck pain, headache, difficulty finding words, irritability, and low back pain.

SF-36 PCS and MCS are presented by group in Figures 1E,F. The average quality of life for participants with PTLDS was considerably worse than the population mean for physical (33.9 ± 9.7, *p* < 0.001) and mental (42.9 ± 10.1, *p* < 0.001) components, with a higher proportion worse than the population mean of 50 for both subscales compared to controls (PCS: 93.4 vs. 23.1%, *p* < 0.001, MCS: 73.8 vs. 23.1%: *p* < 0.001).

### CDC Confirmed Cases vs. Probable Cases

Finally, participants with PTLDS were separated into two groups based on whether their initial presentation met the CDC case definition for LD-confirmed or LD-probable (26), and these groups were compared on all previously reported variables. There were no demographic differences by group, and among the variables in Table 2, only median time from illness onset to treatment (14 days LD-confirmed vs. 7 months LD-probable, *p* = 0.004) and delayed or misdiagnosis (50.0% LD-confirmed vs. 79.0% LD-probable, *p* = 0.033) were statistically significant. Among the variables in Table 3, only the proportion IgG-WB seropositive differed by group (31.7% LD-confirmed vs. 68.4% LD-probable, *p* = 0.008), although this was expected as the LD-probable but not the LD-confirmed group was required to have prior serologic evidence of exposure at study enrollment. Among the standardized questionnaires, the LD-probable group had worse median scores for sleep quality (PSQI; 12.5 vs. 9.0, *p* = 0.031), pain (SF-MPQ; 18.0 vs. 9.0, *p* = 0.021), and depression (18.0 vs. 12.0, *p* = 0.034). However, among the list of 36 current symptoms, the LD-probable group endorsed higher severity of difficulty finding words (*p* = 0.017) and irritability (*p* = 0.027) only.

## Discussion

This study represents the first to compare patients with rigorously defined PTLDS to non-Lyme infected controls on a range of clinical, laboratory, symptom, and quality-of-life parameters. We found that physical exam and clinical laboratory tests showed few abnormalities. However, standardized questionnaires revealed that these patients are highly symptomatic, with clinically significantly poorer quality of life compared both to healthy controls and the US population. These findings were consistent regardless of whether participants met criteria for the initial LD-probable or LD-confirmed group. There was some evidence for greater initial misdiagnosis and more severe current symptoms among the LD-probable group.

Results from the physical exam and laboratory testing in our sample of patients with PTLDS did not show a pattern of significant objective abnormalities. The most notable exception was the higher rate of diminished vibratory sensation on physical exam among participants with PTLDS (32.2%), compared to the 5% expected to fall below the age-adjusted cutoffs used in this analysis (29). This abnormality were not only present among those with a prior diagnosis of neurologic Lyme disease or a later diagnosis of neuropathy but also among those without a specific prior neurologic diagnosis. This finding was also observed in a previous population-based study, in which decreased vibratory sense was noted in 29% of a sample of individuals with prior Lyme disease, and was one of the sole physical exam findings to differ significantly from controls (17). Additionally, although only found in a small subset of our sample (3.4%), two participants met criteria for postural orthostatic tachycardia syndrome, an autonomic condition that has been previously reported following Lyme disease (37). Laboratory tests, including complete blood count, complete metabolic count, and C-reactive protein, showed no significant difference between participants with PTLDS and controls. The rates of current two-tier IgG-WB positivity among those with confirmed early (7/36, 19.4%) and late (4/6, 66.7%) Lyme disease were similar to those found in a 10- to 20-year follow-up study of treated patients assessed to have good overall health (22), further emphasizing that current serology cannot be used as a diagnostic marker for PTLDS.

By contrast, participants with PTLDS reported prominent symptoms that are often diverse and can be moderate to severe in nature. We expected that current symptom severity of fatigue, pain, and cognitive complaints would be higher among PTLDS participants than controls, given that presence of at least one of these symptoms was included in both the IDSA proposed case definition and our enrollment criteria. Indeed, severe levels of each of these were reported by 50.0 (30/6), 28.3 (17/60), and 23.3% (14/60) of participants with PTLDS, respectively, compared to none of the controls for each. Standardized questionnaire scores on fatigue and pain for these participants were also considerably worse than clinically relevant cutoffs (FSS: 55.9 ± 5.9; SF-MPQ: 15.9 ± 6.9; *p* < 0.001 for both). Previous studies have suggested that fatigue and cognitive symptoms are common, and may be particularly important contributors to decreased functioning in this syndrome (38, 39). As Lyme disease incidence continues to increase in endemic regions and spread to new geographic areas (2), PTLDS is likely to be increasingly relevant in the differential diagnosis of patient-reported symptoms commonly encountered by the general internist.

Participants with PTLDS also reported significantly higher severity of an additional 19 diverse symptoms that were not part of the IDSA criteria but which could be diagnostically and clinically relevant. Among these, sleep difficulty was the most frequently reported current symptom, and the symptom with the highest difference in proportion reporting “moderate” or above between cases and controls, suggesting that it may be a clinically important component of this syndrome. Sleep difficulty has been previously identified in the context of Lyme disease (40), and was found to correlate highly with fatigue in one study (17). In our sample, severe sleep difficulty was reported by 31.7% (19/60) of participants with PTLDS and none of the controls, and their PSQI scores were significantly worse than the clinically relevant cutoff for poor sleep quality (PSQI: 14.0 ± 3.5, *p* < 0.001). Additionally, severe visual clarity issues and photophobia were reported by 5.0 (3/60) and 8.3% (3/60) of our sample of participants with PTLDS respectively, compared to none of the controls. Although ophthalmologic signs can occur in untreated disease, persistent subjective symptoms have not been described extensively in PTLDS (41, 42). Finally, those symptoms not statistically significantly different between participants with PTLDS and controls notably included objective signs of rheumatologic, neurologic, or ocular involvement (such as joint swelling, drooping facial muscle, and double vision) that traditionally distinguish untreated Lyme disease from the posttreatment phase (21, 43).

We found further evidence that the physical and mental health-related quality of life of patients with PTLDS is often lower than both controls and US population means. Poor health-related quality-of-life baseline values were previously reported in both US and European treatment trials among patients with persistent symptoms following Lyme disease (1.5–2 SD below the PCS population mean and 0.5–1.5 SD below the MCS population mean) (23, 44). As Klempner and colleagues note, these low scores are comparable to patients suffering from congestive heart failure or osteoarthritis (23). They are also consistent with results from our sample, with more than half of the participants with PTLDS found to have SF-36 PCS that were 1.5 SD below the population mean, and 41.0% had MCS that were 1 SD below the population mean. Scores below these cutoffs were not found among any of the controls.

Delayed or misdiagnosis of early Lyme disease has been hypothesized by some to be less common in recent decades as knowledge of appropriate treatment regimens and serologic testing methods has increased (45). However, as identified in this study and in our previous chart review (8), these risk factors for PTLDS may still be part of the community landscape of Lyme disease diagnosis and treatment. In the current study, we found evidence of exposure to non-recommended antibiotics (such as sulfamethoxazole or cephalexin), steroids, or an extended duration of greater than 30 days from illness onset to initiation of recommended treatment in 59% of the sample of participants with PTLDS. Notably, the rate of delayed or misdiagnosis was also significantly higher among those with initial LD-probable compared to LD-confirmed presentations (79 vs. 50%, *p* = 0.033). We hypothesize that this is due to the non-specific nature of the symptoms in the LD-probable group, which may be more frequently overlooked or attributed to viral illnesses.

Although our study sample was largely recruited from a subset of those referred to an academic referral research center and clinic for PTLDS, our participants’ initial Lyme disease was diagnosed and treated in a range of clinical settings. We chose a retrospective design in the current study in order to capture this heterogeneity, and to capitalize on a more representative and generalizable view of PTLDS in the community setting. Participants included in prospective studies are often ideally and uniformly treated, and limited to specific clinical presentations by design (46, 47). We hypothesize that such factors may contribute to discrepant findings in symptom severity between prospective studies, which tend to report milder residual symptoms following treatment (46, 47), and community-based retrospective studies such as ours and others (16, 17) that describe a higher symptom burden with more significant impacts on quality of life (48).

Our study is limited by the fact that no definitive biomarker exists either for *B. burgdorferi* infection or PTLDS. As such, we cannot be completely certain that our participants with PTLDS had initial signs and symptoms that were correctly attributed to Lyme disease at illness onset. To minimize this risk, we required medical record documentation to establish the initial signs and symptoms, diagnosis, and treatment for Lyme disease that adhered to the IDSA proposed case definition, which few prior studies have done. We also cannot be completely certain that these symptoms are attributable to PTLDS and not other co-morbidities, as was suggested by a recent publication (49). To address this potential confounding effect, our study compared participants with PTLDS to a healthy control group recruited following the same exclusionary criteria, which included a range of conditions independently associated with fatigue, pain, and cognitive dysfunction. Furthermore, while the inclusion of LD-probable patients could also presumably lower specificity of our findings, our analysis did not find a prominent pattern of difference between LD-probable and LD-confirmed groups. Lastly, our study is limited by an inability to address the possibility of recall bias for those variables which were based on self-report and not medical record review.

Our comprehensive case series suggests that the patient-reported symptoms of PTLDS and their effect on life functioning can be clinically significant and long-lasting, regardless of initial Lyme disease presentation. In the absence of a diagnostic biomarker, this study shows that PTLDS can be successfully characterized using a systematic and standardized approach to patient evaluation. This includes special attention to identifying a medical history of initial missed or delayed diagnosis of Lyme disease, which are potentially important risk factors for the development of PTLDS. We speculate that the pattern of symptoms identified in this study could be a tool for the diagnosis of PTLDS, and that well-validated symptom surveys could be used in the future to both identify and monitor treatment progress in patients with PTLDS. Physicians practicing in Lyme disease-endemic areas need to remain alert for the diagnosis of PTLDS when evaluating patients with otherwise unexplained symptoms and prior Lyme disease.

Further research is needed to delineate specific risk factors for PTLDS and the mechanisms of illness in this group of patients in order to guide improvements in diagnostic specificity and treatment options. Biosamples from this cohort of participants will be an important resource for future investigations into both the identification of molecular biomarkers of disease and addressing potential underlying biological mechanisms in PTLDS. Until then, the symptoms of PTLDS, which can be severe and significantly impact quality of life, need to be more effectively identified, validated, and managed as part of integrated patient care. As the prevalence of PTLDS will continue to rise, there will be an increasing need for physician education toward this end.

## Ethics Statement

This study was approved by the Institutional Review Board of the Johns Hopkins University School of Medicine and written consent was obtained from all participants. All subjects gave written informed consent in accordance with the Declaration of Helsinki. The protocol was approved by the Institutional Review Board of the Johns Hopkins University School of Medicine.

## Author Contributions

AR, JA, KB, and MS contributed to the conception of the work. JA, CN, AR, and EM screened patients and assessed eligibility. EM and AR conducted patient interviews and administered surveys. CN and JA performed physical examinations. TY and AR performed the data analysis. EM created all figures. JA, AR, and TY drafted the manuscript. CN, EM, KB, and MS critically revised the paper. All authors have approved the final version and have agreed to be accountable for all aspects of the work.

## Conflict of Interest Statement

The authors declare that the research was conducted in the absence of any commercial or financial relationships that could be construed as a potential conflict of interest.
